# Visual-Semantic Tuning across the Cortex Shifts between Tasks

**DOI:** 10.1523/ENEURO.0183-26.2026

**Published:** 2026-09-08

**Authors:** Tianjiao Zhang, Jack L. Gallant

**Affiliations:** ^1^Helen Wills Neuroscience Institute, University of California, Berkeley, California 94720; ^2^Department of Neuroscience, University of California, Berkeley, California 94720

**Keywords:** fMRI, naturalistic tasks, tuning shift, visual semantics

## Abstract

Attention is a powerful mechanism that dynamically optimizes brain representations to prioritize behaviorally relevant information. While previous studies focusing on single tasks have demonstrated that attention shifts tuning toward attended targets, real-world behavior often requires humans to switch between tasks with different demands. How does visual-semantic tuning in the human brain shift to support the behavioral demands of different naturalistic tasks? To investigate this question, we used voxelwise encoding models to explore how visual-semantic tuning across the human cerebral cortex shifts between two naturalistic human fMRI experiments that used distinct tasks, movie-watching (five male participants) and active navigation (six participants, three male, three female). Results show that visual-semantic tuning in the cortex differed substantially between experiments. Principal component analysis reveals that during navigation, tuning shifts increase the representation of vehicles and traffic signs compared with movie-watching and that these shifts are localized to distinct functional networks. These tuning shifts reconfigure the human brain’s representations of object categories based on their behavioral relevance. These findings suggest that visual-semantic tuning in the brain dynamically shifts toward task-relevant information across naturalistic tasks, optimizing functional representations to achieve diverse behavioral goals.

## Significance Statement

In real-world situations, we switch between many tasks with behaviorally distinct goals that require us to attend to different targets. Here we show that visual-semantic tuning across the human cortex shifts between passive movie-watching and active navigation. These tuning shifts increase the representation of behaviorally relevant objects, such as cars during navigation. Tuning shifts toward different object categories are distributed across distinct functional networks, suggesting that they engage different cognitive mechanisms. These results indicate that visual-semantic tuning in the human brain is highly task-dependent and is optimized to represent behaviorally relevant information.

## Introduction

Attention is a powerful mechanism that optimizes brain function to prioritize processing behaviorally relevant information. Neurophysiology studies in nonhuman primates have demonstrated that attentional effects are found across the visual hierarchy. Attention modulates the gain of neural firing rates as early as the primary visual cortex (V1; McAdams and Maunsell, 1999) and also the baseline of neural firing rates in V2 and V4 (Motter, 1993; Luck et al., 1997; McAdams and Maunsell, 1999). Furthermore, spatial attention causes the receptive fields of neurons in both V4 and MT to shift toward the attended location (Connor et al., 1997; Womelsdorf et al., 2006), and feature-based attention shifts the tuning of V4 neurons toward the attended feature (David et al., 2008). Finally, tuning for visual features in prefrontal neurons changes by task context (Asaad et al., 2000; Johnston and Everling, 2006; Warden and Miller, 2010; McKee et al., 2014).

Complementing these neurophysiological findings, neuroimaging studies in humans have found that attention changes voxel tuning across much of the cerebral cortex. Spatial attention shifts population receptive fields across the visual hierarchy (Klein et al., 2014; Vo et al., 2017). Attention to high-level visual features such as object category, action category, or affordances shifts tuning in lateral occipital (Çukur et al., 2013; Kaiser et al., 2016), parietal (Çukur et al., 2013; Bracci et al., 2017), and prefrontal regions (Çukur et al., 2013; Bugatus et al., 2017; Nastase et al., 2017; Shahdloo et al., 2022). Together, these converging neurophysiology and neuroimaging results suggest that attention acts as a matched-filter mechanism that enhances the representation of task-relevant information and which consequently diminishes the representation of task-irrelevant information (Mazer and Gallant, 2003; David et al., 2008).

Because different tasks have distinct constraints and behavioral goals, attentional optimization of brain representations is likely to be most prominent when comparing across distinct tasks. Indeed, primate neurophysiology studies have reported that different tasks evoked neuronal tuning shifts in many brain regions, including the inferior temporal cortex (Koida and Komatsu, 2007) and the prefrontal cortex (Asaad et al., 2000; Johnston and Everling, 2006; Warden and Miller, 2010; McKee et al., 2014). However, most human studies have only examined attentional modulation of functional representations within the context of a single task. For example, in a detection task participants might be directed to attend to different screen locations or to search for different object categories, while the general stimulus and task structure remains fixed (Harel et al., 2014; Bracci et al., 2017). Thus, attentional effects across multiple tasks remain poorly studied in humans (Kay et al., 2023).

**Figure 1.**
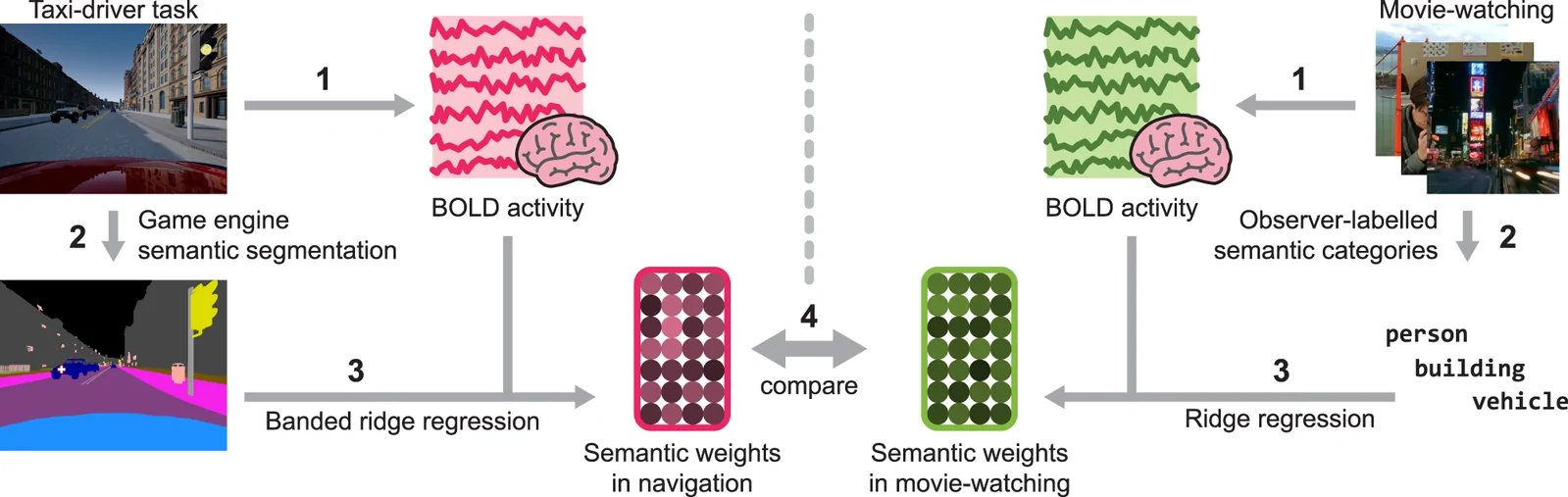
Comparing visual-semantic tuning across tasks. Attention is a powerful mechanism for increasing the brain's efficiency for processing task-relevant information. By operating as a matched-filter mechanism that shifts neural tuning toward targets (Mazer and Gallant, 2003; David et al., 2008), attention dynamically increases the brain's representation of attentional targets. Because attentional demands differ across task contexts, we explored how visual-semantic tuning may differ across naturalistic tasks. To do so, we compared visual-semantic tunings of the brain between a navigation experiment and a movie-watching experiment. In the movie-watching experiment, participants watched clips of various naturalistic movies and videos. To create visual-semantic labels for the movies, each scene was semantically labeled by two independent observers. In the navigation experiment, participants drove through a large virtual city from a first-person perspective and were cued to drive to different locations in the virtual city. To create visual-semantic labels for the stimulus, the game engine was used to compute a ground-truth semantic segmentation of the video. Brain activity was recorded with fMRI in both experiments. Voxelwise encoding models were then fit separately for the subjects in the two experiments to capture the semantic tuning of the brain during navigation and movie-watching. In the driving experiment, the visual-semantic models were fit together with 37 other feature spaces listed in Extended Data Figure 1-1. In the movie-watching experiment, the visual-semantic models were fit together with motion-energy features. Extended Data Figures 1-2 and 1-3 show visual-semantic encoding model performances in movie-watching and driving, respectively. The visual-semantic tuning of the brain in the two experiments was then compared with determine the visual-semantic tuning differences between movie-watching and navigation.

To investigate this gap, we explored how visual-semantic tuning in the cerebral cortex differs between a movie-watching experiment and a navigation experiment (Fig. 1). Here, visual-semantic tuning refers to the response profile of each voxel across object categories, as estimated by encoding model weights. In the movie-watching experiment, one group of participants watched naturalistic videos at fixation (Huth et al., 2012). In the navigation experiment, a different group of participants drove a virtual car from a first-person perspective through a virtual city (Zhang et al., 2025). Brain activity was recorded with fMRI in both experiments. We used voxelwise modeling to characterize visual-semantic tuning across the cortex in both experiments (Naselaris et al., 2011). Because the two experiments were not designed as matched conditions and differ in many respects beyond task, this study is exploratory rather than a controlled test of attentional modulation. Comparison of the visual-semantic model weights obtained in the two experiments nevertheless revealed significant tuning shifts, suggesting the brain shifts its visual-semantic tuning to optimize for the representation of task-relevant categories.

10.1523/ENEURO.0183-26.2026.f1-1 Figure 1-1 Table listing the additional feature spaces included in the navigation task. The name and a short description are given for each feature space. Download Figure 1-1, DOCX file.

**Figure 2.**
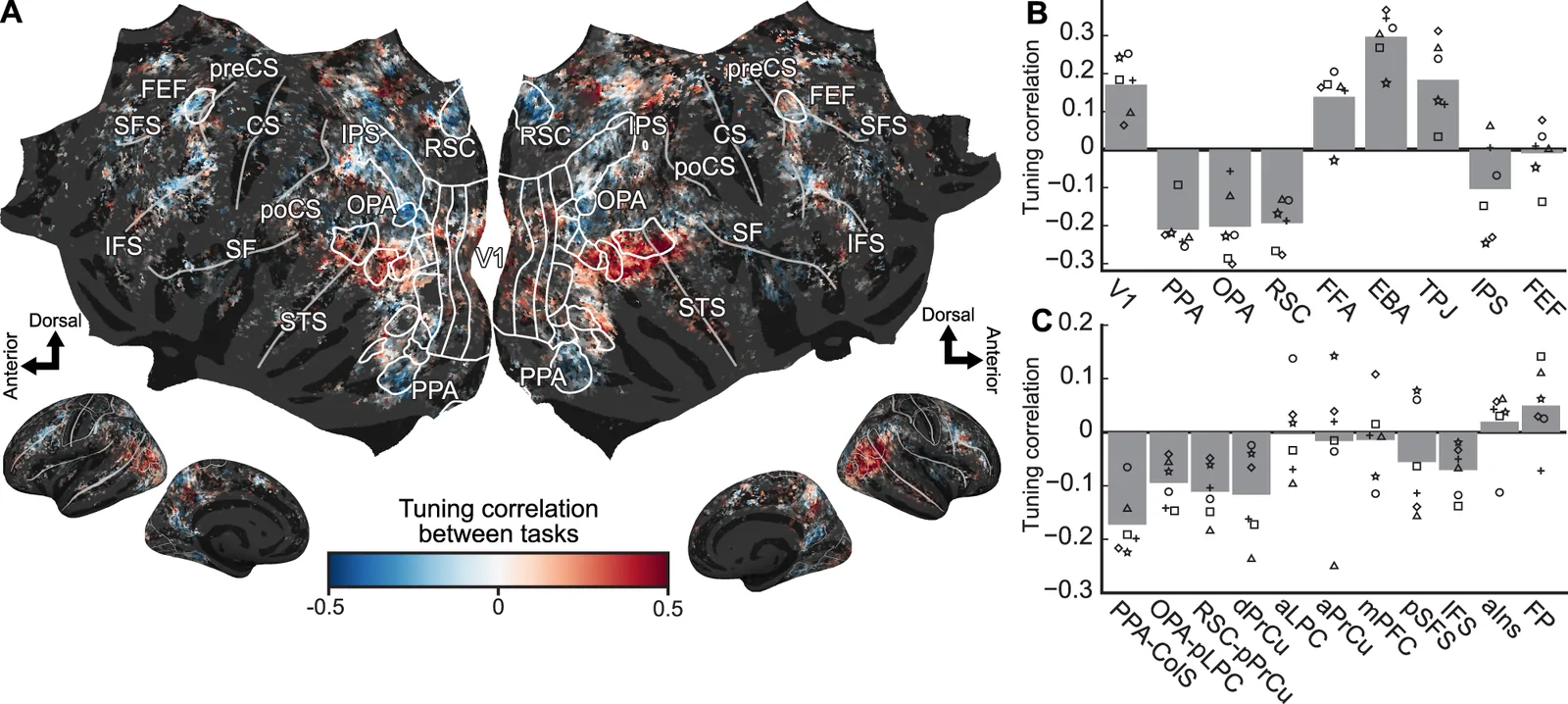
Visual-semantic tuning across the cortex shifts between movie-watching and navigation. Different task demands may cause the brain to shift its visual-semantic tuning between movie-watching and navigation. ***A***, The magnitude of the tuning shift was quantified as the correlation between the visual-semantic tunings in the two experiments at each vertex on the brain. A higher correlation corresponds to a weaker tuning shift and a lower correlation to a stronger tuning shift. The tuning in each navigation participant was compared with the mean tuning in the movie-watching participants. Results show that visual-semantic tuning shifts significantly between tasks in 42.0 ± 6.3% of the cortex (mean ± SD across navigation participants, *p* < 0.05, FDR-corrected bootstrap test). Significant tuning shifts are shown on the group-level map. For correlations in individual navigation participants to the movie-watching mean, see Extended Data Figure 2-1. ***B***, Because movie-watching and navigation likely engage different functional networks, the visual-semantic tuning shifts may differ across functional regions. The average tuning correlation between the two tasks is shown for selected ROIs. Markers indicate individual navigation participants. The tuning shifts are the strongest in PPA, OPA, and RSC, three scene-selective regions, followed by IPS and FEF, two visual attention regions. The tuning shifts are the weakest in FFA, EBA, and TPJ, which are people and socially selective, which are comparable to the tuning shift in V1. These results suggest that while human-selective regions may have more consistent visual-semantic tuning across tasks, visual attention regions and place-selective regions may more flexibly adapt to task demands. ***C***, In other work we had shown the functional network mediating active navigation is also selective for visual-semantics. Here, we examined how visual-semantic tuning in these regions differed between movie-watching and navigation. Bars show the group-average correlation in visual-semantic tuning between the experiments in each region, and markers indicate individual subjects. All but two regions show a zero or negative correlation in visual-semantic tuning between experiments, suggesting that the navigation network flexibly shifts its visual-semantic representations across tasks. These results show that the spatial navigation network, like visual attention regions, flexibly shifts its visual-semantic tunings when engaged during navigation.

10.1523/ENEURO.0183-26.2026.f1-2 Figure 1-2 Visual semantic model performance in movie-watching. Voxel are colored by the split R^2^ model performance for the visual semantic model. The group-average performance is shown at the top on the fsaverage surface, and individual subjects are shown below the group average. Visual semantics explains activity across the anterior visual cortex, including RSC, IPS, OPA, MT, LO, FFA, and PPA, and also parietal regions such as the precuneus and TPJ, and also parts of the precentral sulcus. Download Figure 1-2, TIF file.

**Figure 3.**
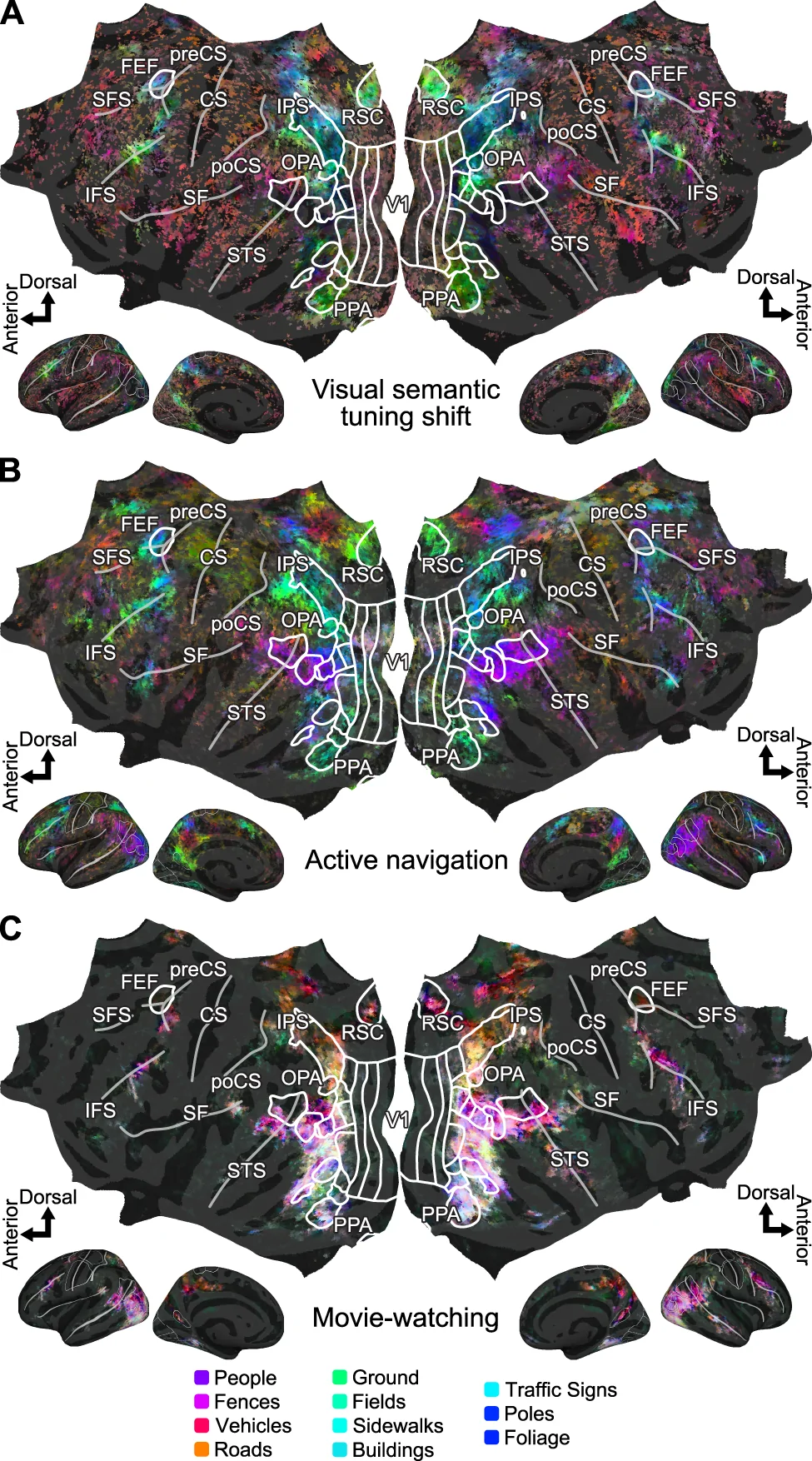
Visual-semantic tuning shifts vary across the cortex. While the correlation analysis shows that visual-semantic tuning differed between the two tasks, it does not reveal how the cortex reorganizes its selectivity across semantic categories. To identify the specific differences in visual-semantic selectivity between movie-watching and navigation, we subtracted the visual-semantic weights during movie-watching from the visual-semantic weights during navigation at each vertex on the brain. While there were some stimulus statistics differences between the two experiments (Extended Data Fig. 3-1), they did not explain any significant amounts of variance in these tuning differences (11.4 ± 2.8% mean ± SD across participants, chance = 9.9%, *p* > 0.05, permutation test). Principal components analysis identified three significant group-level PCs in these 11-dimensional tuning shift vectors (*p* < 0.05, permutation test; Extended Data Fig. 3-2). To visualize these tuning shifts, we projected the tuning shifts on to these three PCs, which are mapped to the red, green, and blue color channels, respectively. To aid in interpretation, the 11 visual-semantic categories were also projected into this space, and their colors are shown at the bottom. ***A***, Visual-semantic tuning shifts between active navigation and movie-watching are shown on the group-level map (for individual navigation participants, see Extended Data Fig. 3-3). The IPS and FEF show a shift toward sidewalks, traffic signs, and buildings. The PPA, RSC, and inferior frontal sulcus show a shift toward ground and fields. The rest of the cortex, including precuneus, TPJ, insula, and prefrontal cortex, show a shift toward vehicles. Extended Data Figures 3-4 and 3-5 show the average tuning shifts within selected ROIs. ***B***, ***C***, For comparison, the visual-semantic tuning in navigation and movie-watching were also projected into this space. These maps reveal a striking difference in visual-semantic tuning across the cortex between tasks and show that tuning shifts vary systematically across ROIs between naturalistic tasks.

10.1523/ENEURO.0183-26.2026.f1-3 Figure 1-3 Visual semantic model performance in driving. Voxel are colored by the split R^2^ model performance for the visual semantic model. The group-average performance is shown at the top on the fsaverage surface, and individual subjects are shown below the group average. Visual semantics explains activity across the anterior visual cortex, including RSC, IPS, OPA, and PPA, and also parietal regions such as the TPJ. Additionally, the visual semantic model also explains activity in multiple prefrontal regions outside known ROIs, including the posterior medial PFC and along the SFS and IFS, suggesting that the representation of visual semantic information may be more distributed across the cortical surface during driving than movie-watching. Download Figure 1-3, TIF file.

## Materials and Methods

In this study, we compared visual-semantic tuning in the human cerebral cortex in data collected from two experiments. Participants in the first experiment watched movies, while participants in the second experiment engaged in active navigation. Voxelwise modeling was used to recover visual-semantic tuning in both experiments, for every cortical voxel in every participant. Because the data from the movie-watching and navigation experiments were collected from different participants and exist in different anatomical spaces, FreeSurfer was used to map data across anatomical spaces of the participants.

### Participants

Five participants (male, ages 25–32) participated in the movie-watching experiment, and six participants (three male, three female, ages 24–34) participated in the navigation experiment. All participants were healthy adults with normal or corrected-to-normal vision. The experimental procedures were approved by the Institutional Review Board at the University of California, Berkeley, and written informed consent was obtained from all participants.

### Movie-watching experiment

#### Task

The movie data was collected in previously published datasets (Nishimoto et al., 2011; Huth et al., 2012). Briefly, natural movies were drawn from the Apple QuickTime HD gallery and YouTube and cropped to 512 × 512 pixels. Multiple 10–20 s clips were concatenated to form 21 10 min runs. Twelve runs were used as model training runs, and nine runs were used for model validation. Five male participants (ages 25–32) watched these movies at fixation while BOLD activity was recorded.

Video clips were drawn from the following movies: *Australia*, *Bolt*, *Bride Wars*, *Changeling*, *Duplicity*, *Fuel*, *Hotel for Dogs*, *Inkheart*, *King Lines*, *Mall Cop*, *Madagascar 2*, *Pink Panther 2*, *Proud American*, *Role Models*, *Sharkwater*, *Star Trek*, *The Tale of Despereaux*, *Warren Miller’s Higher Ground*, and *Yes Man*. Clips were also taken from the Artbeats HD, BBC Motion Gallery, Mammoth-HD, and The Macaulay Library. Additional clips were taken from YouTube: IGN game of the year 2008, JAL Boeing 747 landing Kai Tak, The American recovery and reinvestment plan, and Where the hell is Matt?.

#### Feature spaces

To create visual-semantic features, an observer manually tagged every second of the movies with WordNet labels (Miller, 1995). Categories were tagged if they appeared in at least half of each second. One-hot encoding features were then constructed for all categories that appeared in the movies. Additionally, features were also included for all superordinate categories for the labeled categories. For example, in addition to “dog,” features were also included for “canine” and “domestic animal.” There were 1,364 object categories and 341 superordinate categories, for a total of 1,705 features.

Because the high-level visual-semantic content and the low-level visual structure of naturalistic movies are correlated, models fit to only the visual-semantic features may be confounded by stimulus correlations between high- and low-level features. To account for these stimulus correlations in subsequent analyses, motion-energy features (Nishimoto and Gallant, 2011) were also computed for these movies to capture their low-level visual structure. To create this feature space, the screen was tiled with 6,555 spatiotemporal quadrature Gabor filters of varying size, orientation, and temporal frequency, and the output of the filters applied to the movie was used as features.

### Navigation experiment

#### Task

The navigation data were collected in Zhang et al. (2025). Briefly, participants performed a taxi-driver task in a virtual city while brain activity was recorded with fMRI. A large dynamic virtual city was constructed with Unreal Engine 4 and CARLA (Dosovitskiy et al., 2017) and was populated with both vehicular and pedestrian traffic. Participants used a custom MR-compatible steering wheel and pedal set to drive a car through their virtual world from a first-person perspective. Prior to scanning, participants learned the layout of the world. In the scanner, the taxi-driver task was divided into trials. On each trial, a participant was cued to navigate to a particular location in the world. The participants were instructed to use their knowledge of the world to drive to the cued location via the quickest path, while respecting all traffic rules. After the participant arrived at the destination, a new trial began. Participants were allowed to freely view the screen while driving, and eyetracking data were collected at 60 Hz. Trials were recorded with Unreal Engine's built-in demo recording system. Data were collected in 11 min runs. A total of 120 min of data was collected from Participant 1 in two sessions, and 180 min of data each was collected from Participants 2–6 in three sessions.

#### Feature spaces

To compute visual-semantic features, the game engine was used to semantically segment the participant's view to determine the category of the object rendered at each pixel. This semantic segmentation covered 16 categories of 3D assets used to create the virtual environment: buildings, people, vehicles, roads, road lines, sidewalks, traffic signs, poles, walls, fences, fields, ground, foliage, self, miscellaneous objects, and sky. Visual-semantic categories were also included for on-screen display items that were overlaid on the screen rather than rendered in-world: the “go to” and “arrived” cues and the speedometer. The visual-semantic feature space also included 14 additional categories for objects and on-screen display items that appeared during the learning phase, but not during data collection.

Because participants are unlikely to allocate equal attention to all areas of the screen, the visual-semantics of the entire screen may not accurately reflect representations of visual-semantics with the influence of top-down attention. Therefore, the gaze location was used as a proxy of how participants allocated attention. For each sample in the eyetracking data, a circle of 5° diameter was drawn around the gaze position on the corresponding semantic segmentation frame. Then the fraction of this circle that was occupied by each of the 16 categories was calculated and used as features.

In the navigation experiment, the visual-semantic content of the scene covaries with many other features, such as the low-level visual structure of the scene, the participants' motor actions, and other navigation-related parameters. The contribution of the representation of these other features to brain activity was captured by 37 additional feature spaces [see Extended Data Fig. 1-1 for a list and Zhang et al. (2025) for detailed descriptions of each feature space]. In total, these 37 feature spaces encompassed 28,101 additional features.

### Voxelwise encoding models

Voxelwise modeling (Naselaris et al., 2011; Nishimoto et al., 2011; Huth et al., 2012, 2016; Deniz et al., 2019; Lescroart and Gallant, 2019; Nunez-Elizalde et al., 2019) was used to quantify visual-semantic tuning across the brain in each of the two experiments. VM models the time series of the BOLD activity in each voxel as a linear combination of the time series of all features from all feature spaces. In the movie-watching experiment, we fit models for the 1,705 WordNet visual-semantic features and 6,555 motion-energy features to the BOLD activity of each voxel. In the navigation experiment, we fit models for the 16 visual-semantic features along with 28,101 other features in 37 additional feature spaces. To compensate for differences in the size of feature spaces and for correlations between features, banded ridge regression was used to estimate the optimal model weights (Nunez-Elizalde et al., 2019; Dupré la Tour et al., 2022). To capture the hemodynamic response, an FIR filter was implemented by including delayed copies of all features at five temporal delays. Data in each participant were partitioned by holding out the last run in each session as the test set and using the remaining runs for training. The optimal model weights and shape of the FIR filter for each voxel were empirically selected by cross-validation on the training set. Model performance was then quantified by the coefficient of determination (*R*^2^) between the predicted and actual brain activity on the test set. Permutation tests were used to estimate statistical significance in each voxel. A significance mask was determined at an FDR-corrected *p* < 0.05 level in each participant. For each voxel, the joint model performance for all models was then partitioned over models for each of the feature spaces (two in the movie-watching experiment and 38 in the navigation experiment). Here we call these values the split *R*^2^ scores. This study focuses on the visual-semantic models fit in the two experiments (for visual-semantic model performances, see Extended Data Figs. 1-2, 1-3).

To recover the visual-semantic tuning of each voxel in each experiment, the fit model weights were averaged across the five delays. However, because banded ridge regression optimizes the regularization parameter for each feature space in each voxel, the scale of the model weights differs across voxels. To account for this scaling difference, the recovered vectors were first normalized to have unit norm and then scaled by the split *R*^2^ score of the visual-semantic model. This rescaling effectively corrects for the differences in the scales of model weights caused by different regularization hyperparameters.

### Localizers for known functional ROIs

Separate functional localizer experiments were run to identify conventional functional ROIs in each participant. ROIs were determined using five sets of localizers: a retinotopic localizer, an MT localizer, a visual category localizer, a motor localizer, and a theory-of-mind localizer.

#### Retinotopic localizer

Retinotopic localizers were collected in four 9 min runs. In two runs, rotating wedges were used to determine visual angles. In the other two runs, expanding rings were used to determine eccentricity. Visual angle and eccentricity maps were then used to delineate V1, V2, V3, V4, V3A, V3B, and V7 (Hansen et al., 2007).

#### MT localizer

MT localizer data were collected in four 90 s runs. These runs consisted of alternating 16 s blocks of motion-coherent and motion-incoherent random dot fields. The contrast between coherent and incoherent blocks was used to delineate MT+.

#### Visual category localizer

Visual category localizer data were collected in six 4.5 min runs. These runs consisted of 16 blocks of 16 s each. In each block, 20 images of either places, faces, body parts, nonhuman animals, household objects, or phase-scrambled objects were displayed. Each image was displayed for 300 ms followed by a 500 ms blank. The contrast between faces and objects was used to define the fusiform face area (FFA; Kanwisher et al., 1997). The contrast between body parts and objects was used to define the extrastriate body area (EBA; Downing et al., 2001). The contrast between places and objects was used to define the parahippocampal place area (PPA; Epstein and Kanwisher, 1998), occipital place area (OPA; Dilks et al., 2013), and retrosplenial cortex (RSC; Aguirre et al., 1998).

#### Motor localizer

Motor localizer data were collected in one 10 min run. Participants were cued to randomly alternate between six motor tasks in 20 s blocks. For “hand,” “foot,” “mouth,” “speech,” and “rest,” the cue was simply that word presented at the center of the screen. For the saccade condition, participants were shown a screen filled with randomly distributed saccade targets.

In the “hand” blocks, participants were instructed to make small finger-drumming movements. In the “foot” blocks, participants were instructed to make small toe movements. In the “mouth” blocks, participants were instructed to make small lip movements without moving the tongue or jaw. In the “speak” blocks, participants were instructed to continuously subvocalize self-generated sentences without physically speaking. In the “saccade” blocks, participants were instructed to continuously saccade between targets presented on the screen. A linear model was used to find the change in BOLD response in each voxel in each condition relative to the mean BOLD response.

Weights for the hand, foot, and mouth responses were used to delineate the primary motor and somatosensory areas for hands (M1H, S1H), feet (M1F, S1F), and mouth (M1M, S1M). Weights for the saccade responses were used to delineate the intraparietal sulcus (IPS), frontal eye fields (FEFs; Paus, 1996), and frontal operculum (FO; Corbetta et al., 1998). Weights for the “speak” responses were used to delineate Broca's area and the superior ventral premotor speech area (sPMv).

#### Theory-of-mind localizer

Theory-of-mind localizer data were collected in two 5 min runs. Participants were shown a series of short stories consisting of false belief, false photograph, desire, physical description, and nonhuman description stories (Saxe and Kanwisher, 2003). Each story was shown for 10 s. After each story, participants were presented with a fill-in-the-blank two-alternative forced choice question for 4 s and responded with a buttonbox. Half of the questions concerned the belief of characters in the stories, while the other half were concerned with factual information. The contrast between belief and factual questions was used to delineate the temporoparietal junction (TPJ).

### Comparison of visual-semantic tuning across tasks

In order to compare visual-semantic tuning in the cerebral cortex between participants in the movie-watching and navigation experiments, we must map between the anatomical spaces of the participants and also between the visual-semantic feature spaces used by the two experiments.

#### Mapping between participants

The data from the movie-watching and navigation experiments were collected from different participants. To compare visual-semantic tuning between participants from the two experiments, the data must first be in the same anatomical space. Because visual-semantic tuning is largely consistent across participants (Huth et al., 2012; Visconti di Oleggio Castello et al., 2025), the simplest approach would be to compare group-average models from both experiments in a template space. Creating a group-average model is well suited for data from the movie-watching experiment, which uses a purely stimulus-driven task with no behavioral variability. However, because the navigation task involves active behavior that varies across individuals, a group-average model is less well suited for this experiment. To preserve individual differences in navigation, we therefore mapped the group-averaged movie-watching data to each individual participant in the navigation experiment, and compared each navigation participant with the group-average movie-watching data.

To map between anatomical spaces, we first mapped the cortical voxels recorded in each movie-watching participant into the fsaverage6 atlas space. This mapping was implemented in several steps. First, the surf2surf function in FreeSurfer (Dale et al., 1999) was used to calculate a mapping from the native space of each movie-watching participant to the fsaverage surface. This mapping was used to project visual-semantic tuning vectors from each movie-watching participant to the fsaverage surface. Then, surf2surf was used to compute a mapping from the fsaverage surface to each navigation participant's surface. The group average movie-watching visual-semantic tuning map was then mapped from fsaverage to the cortical surface of each navigation participant. Finally, the visual-semantic tuning between movie-watching and navigation was compared at each vertex in the native cortical space of each navigation participant.

#### Comparing across visual-semantic feature spaces

The visual-semantic feature space created for the movie-watching experiment consisted of 1,705 semantic features drawn from common WordNet categories. In contrast, the visual-semantic feature space created for the navigation experiment consisted of 16 distinct features defined by the semantic segmentation component in the game engine. Therefore, to assess tuning shifts between experiments, these feature spaces needed to be aligned by defining a shared visual-semantic subspace. Although the WordNet feature space covers a large number of visual-semantic categories, it did not encompass all 16 visual-semantic categories in the navigation experiment. We excluded four visual-semantic categories in the navigation experiment that did not correspond to WordNet categories: “None” labeled untagged objects, “other” served as a catch-all for unlabeled objects, “road lines” represented a driving-specific category absent from movies, and “self” did not appear due to the third-person perspective of movie stimuli. We also excluded “walls” because participants encountered no wall-labeled objects in the virtual environment, preventing tuning estimation despite its presence in both taxonomies. The shared visual-semantic space thus spanned 11 categories: buildings, fences, people, poles, roads, sidewalks, foliage, vehicles, traffic signs, fields, and ground.

Because of the hierarchical nature of WordNet, there was a many-to-one correspondence between the WordNet semantic features in the movie-watching experiment and the disjoint semantic segmentation features in the navigation experiment. For example, the category “Buildings” includes many subordinate categories such as “skyscraper,” “barn,” and “house,” among others. To account for this many-to-one correspondence, the WordNet visual-semantic tuning was averaged across each category with all of its subordinate categories. Doing so produced 11 nonhierarchical categories directly comparable to the visual-semantic categories in the navigation experiment.

### Quantifying the visual-semantic tuning difference between tasks

Once the movie-watching and navigation tuning were anatomically aligned, the tuning differences between the experiments could be evaluated. In prior work we used a tuning shift index (TSI) to quantify such differences (Çukur et al., 2013). However, the TSI measures the strength of tuning shifts along a known axis in semantic space. Because in this study we have no a priori knowledge of what specific visual-semantic categories were attended in either experiment, the TSI is not an appropriate measure. Instead, we quantified both the direction and magnitude of task-related tuning shifts in semantic space. The strength of the tuning shift was quantified as the correlation between the two tuning vectors at each vertex. A correlation of 1 indicates that there is no visual-semantic tuning shift, a correlation of −1 indicates that the vertex shifts to opposite tunings between the two experiments, and a correlation of 0 indicates that the vertex shifts to orthogonal tunings between these two experiments. The direction of the visual-semantic tuning shift was quantified as the difference between the tuning vectors in the two experiments. To account for the difference in weight vector scales between the experiments, the norms of weight vectors in each participant were first separately normalized across the cortex such that the second percentile became 0 and the 98th percentile became 1, and then the normalized movie-watching weight vector at each vertex was subtracted from the navigation weight vector. The resulting vector describes the tuning shift direction at each vertex.

### Bootstrap testing of the significance of tuning differences

A bootstrap procedure was used to test whether any visual-semantic tuning differences between movie-watching and navigation were statistically significant at each vertex in each navigation participant. If visual-semantic tuning at each vertex remained constant across tasks, the correlation between visual-semantic weights from movie-watching and navigation should be close to 1. If visual-semantic tuning differed across movie-watching and navigation, then the correlation should be significantly <1. A bootstrap procedure was used to estimate the distribution of these correlations. To establish a null distribution describing the expected correlation values for when no tuning shifts occur, the movie-watching visual-semantic weights were resampled 1,000 times. On each bootstrap, the data were resampled in 20-TR blocks of the feature and BOLD time series, and the encoding model was refit to this resampled data. The weights from these refit models were then correlated against the original movie-watching weights to form a null distribution for when no tuning shift occurs. The navigation visual-semantic weights were then also bootstrapped 1,000 times using the same procedure. On each iteration, bootstrapped navigation visual-semantic weights were correlated with the movie-watching visual-semantic weights to obtain a distribution of between-task correlations. Statistical significance was determined at each vertex by comparing these two distributions.

### Comparing the representation of object categories between tasks

The analyses above characterized the tuning of individual cortical vertices to different object categories. The aggregate effects of tuning shifts across the cortex may also cause the spatial organization of functional networks to change across tasks. Therefore, we also examined how task context affects the cortical distribution of visual-semantic tuning for each category between the movie-watching and navigation experiments.

The difference in the representation of object categories between the movie-watching and navigation tasks was quantified as the spatial correlation across the cortex for the weight maps for each of the object categories between the two tasks. A correlation of 1 indicates that the object category is represented by the same brain regions, while a correlation of −1 indicates that the object category is represented by the inverse of the set of functional regions between the two experiments, and a correlation of 0 indicates that the object category is represented by orthogonal sets of brain regions between these two experiments. The same bootstrap procedure described above to assess tuning differences was used to determine whether the representation of object categories was significantly different between movie-watching and navigation. On each of the 1,000 bootstraps of the movie-watching weights, we correlated the bootstrapped weights for each object category with the original weights to obtain a distribution of the expected null correlations. Statistical significance was determined for each object category by comparing the actual correlation with this bootstrapped null distribution.

### Animacy index

Because previous experiments had identified the contrast between animate and inanimate objects as a primary organizational principle for object representations (Naselaris et al., 2012; Grill-Spector and Weiner, 2014), we investigated whether the cortex reorganizes object representations along this animacy dimension to reflect their behavioral relevance between tasks. To do so, we defined an animacy index to quantify where the representation of each object category fell along the animate-inanimate spectrum. We first used an earlier study (Naselaris et al., 2012) to identify a list of animate and inanimate objects. Using the movie-watching weights as a reference baseline, we computed template “animate” and “inanimate” representations by averaging the visual-semantic weights separately across all animate categories and across all inanimate categories. The animacy index for any object category was then computed as the difference between its correlation with the animate template and its correlation with the inanimate template, normalized by the correlation between the two templates:

$$\mathrm{animacy}\;\mathrm{inde}x_{\mathrm{category}}\;=\;\frac{\mathrm{corr}(\mathrm{weight}s_{\mathrm{category}},\;\mathrm{templat}e_{\mathrm{animate}})\;-\;\mathrm{corr}(\mathrm{weight}s_{\mathrm{category}},\;\mathrm{templat}e_{\mathrm{inanimate}})}{\mathrm{corr}(\mathrm{templat}e_{\mathrm{animate}},\;\mathrm{templat}e_{\mathrm{inanimate}})}.$$

This animacy index ranges from −1 (fully inanimate representation) to +1 (fully animate representation). The animacy index was then computed for each of the 11 shared visual-semantic categories separately in each participant in the movie-watching and navigation experiments. To determine whether the animacy index of an object category differs between tasks, we used a permutation test. Because each participant constitutes one sample for the representation of each object category, permutation of group membership participants across movie-watching and navigation were used to determine a null distribution for difference in animacy indices.

### Code availability

Supporting code will be made available on GitHub upon publication.

## Results

Prior studies suggest that attention improves task performance by dynamically increasing the representation of task-relevant information in the brain, while decreasing the representation of irrelevant information. However, those studies only examined these effects under a narrow range of highly controlled conditions. Few prior studies have sought to examine how attention might change the representation of information across complex, naturalistic tasks (Kay et al., 2023).

Movie-watching and navigation are two naturalistic tasks that likely require participants to attend to different visual-semantic categories in order to perform each task optimally. Therefore, to examine how attention affects the representation of task-relevant information under naturalistic conditions, we compared visual-semantic tuning across the cerebral cortex in a group of participants who passively watched narrative movies (Huth et al., 2012) versus visual-semantic tuning in a different group of participants who navigated through a virtual world (Zhang et al., 2025). BOLD activity was recorded with fMRI in both experiments, and voxelwise encoding models were used to capture visual-semantic representations in the brain in the two experiments separately.

### Visual-semantic tuning differs between movie-watching and navigation

To determine whether visual-semantic representations and tuning differ between the two tasks, we compared visual-semantic weights obtained from the participants who watched movies to those obtained from participants who navigated in a virtual world. The average visual-semantic weights from the movie-watching participants were mapped to the cortical surface of each navigation participant (see Materials and Methods). For each navigation participant, the visual-semantic weights during navigation at each cortical vertex were compared with the group-average movie-watching weights projected into that participant's anatomical space. Here, a correlation that is significantly <1 indicates that visual-semantic tuning differed between the two tasks. Results show that visual-semantic weight vectors differed significantly across much of the cortex between the two tasks. Between movie-watching and navigation, the correlation was significantly <1 in 42.0 ± 6.3% of cortical vertices (mean ± SD across navigation participants, *p* < 0.05, FDR-corrected bootstrap test; Fig. 2*A*). The correlation between visual-semantic tunings in the two tasks is shown on the flattened group-level cortical surface in Figure 2*A* (for comparisons of individual navigation participants with the movie-watching participants, see Extended Data Fig. 2-1). These results show that there are widespread cortical visual-semantic tuning shifts across tasks.

10.1523/ENEURO.0183-26.2026.f2-1 Figure 2-1 Individual driving subject visual semantic tuning correlations between movie-watching and navigation as in Fig. 2A. Much of the cortex significantly shifts its visual semantic tuning between tasks. Consistent across subjects, RSC, IPS, OPA, and PPA show the strongest shift, while FFA, EBA, and EPJ show the weakest shift. Download Figure 2-1, TIF file.

Because movie-watching and navigation likely engage different functional networks, we sought to determine whether the visual-semantic tuning shifts differ across functional regions. Inspection of Figure 2*A* suggests that the strength of the tuning difference differs systematically across brain regions. To better understand region-specific tuning differences, we determined the average correlation within regions of interest (ROIs) that are known to be attention-related or selective for visual-semantic categories (Fig. 2*B*). Results show that tuning shifts are the strongest in the PPA, OPA, and RSC, followed by the IPS and FEF; tuning differences are weakest in the FFA, EBA, TPJ, and V1. In sum, the weakest shifts were found in human-related and social ROIs (FFA, EBA, and TPJ), and the strongest tuning shifts were found in place-selective (PPA, OPA, and RSC) and visual attention (IPS and FEF) ROIs. These results suggest that while human-selective regions show the most consistent visual-semantic tuning between movie-watching and navigation, place-selective and visual attention regions are more flexible and change their visual-semantic tunings to adapt to the different demands of the two tasks.

### Tuning differences are unlikely to be related to differences in stimulus statistics

One potentially important confound in this study is that the visual-semantic stimulus statistics differed between the movie-watching and navigation experiments. For example, “people” occurred much more frequently in the movie-watching experiment than in the navigation experiment. It is theoretically possible that the observed tuning differences might be an artifact of these differences in stimulus statistics. We used two convergent methods to test that stimulus statistics did not confound our results. First, we computed the relative differences between the two experiments across each of the 11 visual-semantic categories evaluated in this study (Extended Data Fig. 3-1). The amount of variance in the observed tuning shifts that could be explained by stimulus differences between movie-watching and navigation is not significant (11.4 ± 2.8% mean ± SD across participants, chance = 9.9%, *p* > 0.05, permutation test). In the second test we computed the correlation between the stimulus differences and the principal components of the tuning shifts. The stimulus statistic differences were not significantly correlated with any of the PCs of the tuning shifts (*p* > 0.05, permutation test). Taken together, these two analyses suggest that the observed semantic tuning shift between movie-watching and navigation are likely intrinsic to the brain, rather than an artifact of the difference in stimulus statistics in the two experiments.

### Tuning shifts differ across functional regions

While the analyses above show that visual-semantic tuning differed between movie-watching and navigation, they do not reveal which specific visual-semantic categories the brain prioritizes in each task. To characterize visual-semantic targets of the tuning shifts between movie-watching and navigation, we subtracted the average movie-watching weights from the navigation weights at each vertex in every navigation participant, and then applied principal components analysis (PCA) to these tuning differences. Across all six navigation participants, we find three PCs that explain significant amounts of variance in these tuning differences (*p* < 0.05, permutation test), accounting for 53.9 ± 6.69% of the total variance (mean ± SD across navigation participants; Extended Data Fig. 3-2). Figure 3*A* shows these three PCs on the group-level cortical surface, mapped to the red, green, and blue color channels, respectively (for individual navigation participants, see Extended Data Fig. 3-3). Figure 3, *B* and *C*, shows visual-semantic tuning during movie-watching and navigation on these same PCs. Inspection of Figure 3 reveals that the IPS and FEF, two regions that mediate visual attention, shift their visual-semantic tuning toward sidewalks, traffic signs, and buildings during navigation compared with movie-watching. The PPA and RSC, two place-selective regions, and the inferior frontal sulcus shift their visual-semantic tuning toward ground and fields. Many other cortical regions, including precuneus, TPJ, insula, and prefrontal cortex, shift their tuning toward vehicles. (Average tuning shifts in individual ROIs are shown in Extended Data Fig. 3-4.) These results suggest that functional regions separately reconfigure their visual-semantic representations between tasks, likely reflecting complex interactions between their baseline representations and their task-specific roles.

10.1523/ENEURO.0183-26.2026.f3-1 Figure 3-1 Relative stimulus differences between experiments. Categories to the left of the “equal” line appeared more frequently in the navigation experiment, and those to the right of the “equal” line appeared more frequently in the movie-watching experiment. Bars show average frequency ratio over subjects, and dots indicate difference in individual subjects. These stimulus differences are not significantly correlated with any of the tuning shift components (p > 0.05, permutation test), suggesting that the tuning shifts are unlikely to be driven by the stimulus differences. Download Figure 3-1, TIF file.

10.1523/ENEURO.0183-26.2026.f3-2 Figure 3-2 Fraction variance explained by the group-level tuning shift principal components in individual subjects. Closed circles indicate components that explain a significant amount of variance (permutation test, p < 0.05), and open circles indicate those that do not. The first three PCs explain significant amounts of variances in all subjects, suggesting that there are three consistent dimensions in the cortical tuning shift between movie-watching and navigation. Download Figure 3-2, TIF file.

10.1523/ENEURO.0183-26.2026.f3-3 Figure 3-3 Individual subject visual semantic tuning shifts in driving as shown in Fig. 3A. Download Figure 3-3, TIF file.

**Figure 4.**
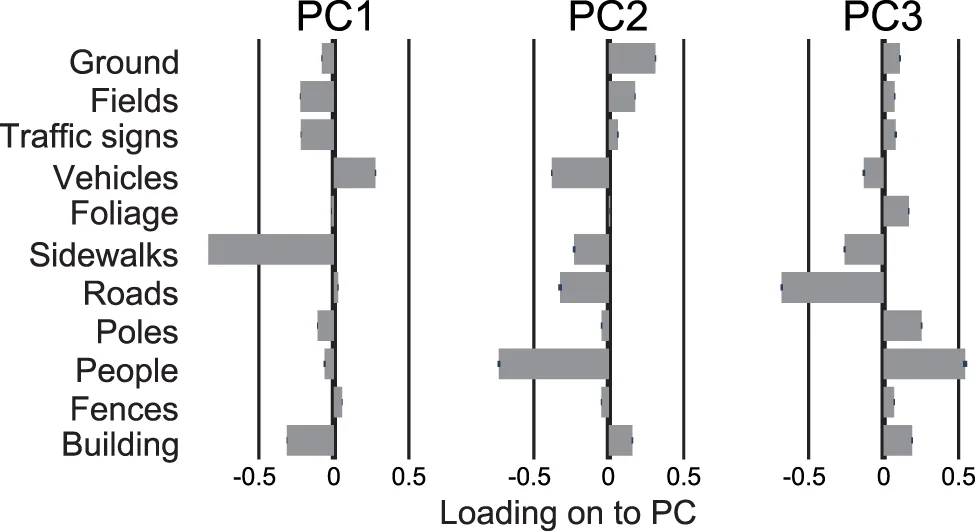
Loadings of the 11 visual-semantic categories onto the three group-level principal components. Tuning shifts serve to increase the representation of task-relevant information across the brain. To understand the visual-semantic dimensions that the brain reprioritizes between navigation and movie-watching, we examined the loadings of the 11 categories on to the three significant group-level principal components (PCs) of the tuning shifts. PC 1 shows an almost singular shift toward vehicles during navigation, and away from sidewalks, buildings, traffic signs, and fields, suggesting that the primary tuning shift enables the brain to better identify other vehicles in the environment. PC 2 shows a shift toward buildings, ground, fields, foliage, and traffic signs, and away from vehicles, roads, sidewalks, and people, suggesting this shift enables the brain to better discriminate between scene identity-relevant categories and the entities that populate the scene. PC 3 shows a shift away from roads, sidewalks, and vehicles, and primarily toward people, suggesting that this shift enables the brain to better discriminate between navigational affordances and people. These PCs suggest that the brain shifts its visual-semantic representations to better represent and differentiate between task-relevant object categories.

10.1523/ENEURO.0183-26.2026.f3-4 Figure 3-4 Average tuning shifts in individual ROIs. The average shift (a.u.) for each category is shown for A) V1, as a comparison, B) the scene-selective regions of PPA, OPA, and RSC, C) people- and socially-selective regions of FFA, EBA, and TPJ, and D) visual attention regions of IPS and FEF. Bars show average shift across subjects, and dots indicate individual subjects. Download Figure 3-4, TIF file.

**Figure 5.**
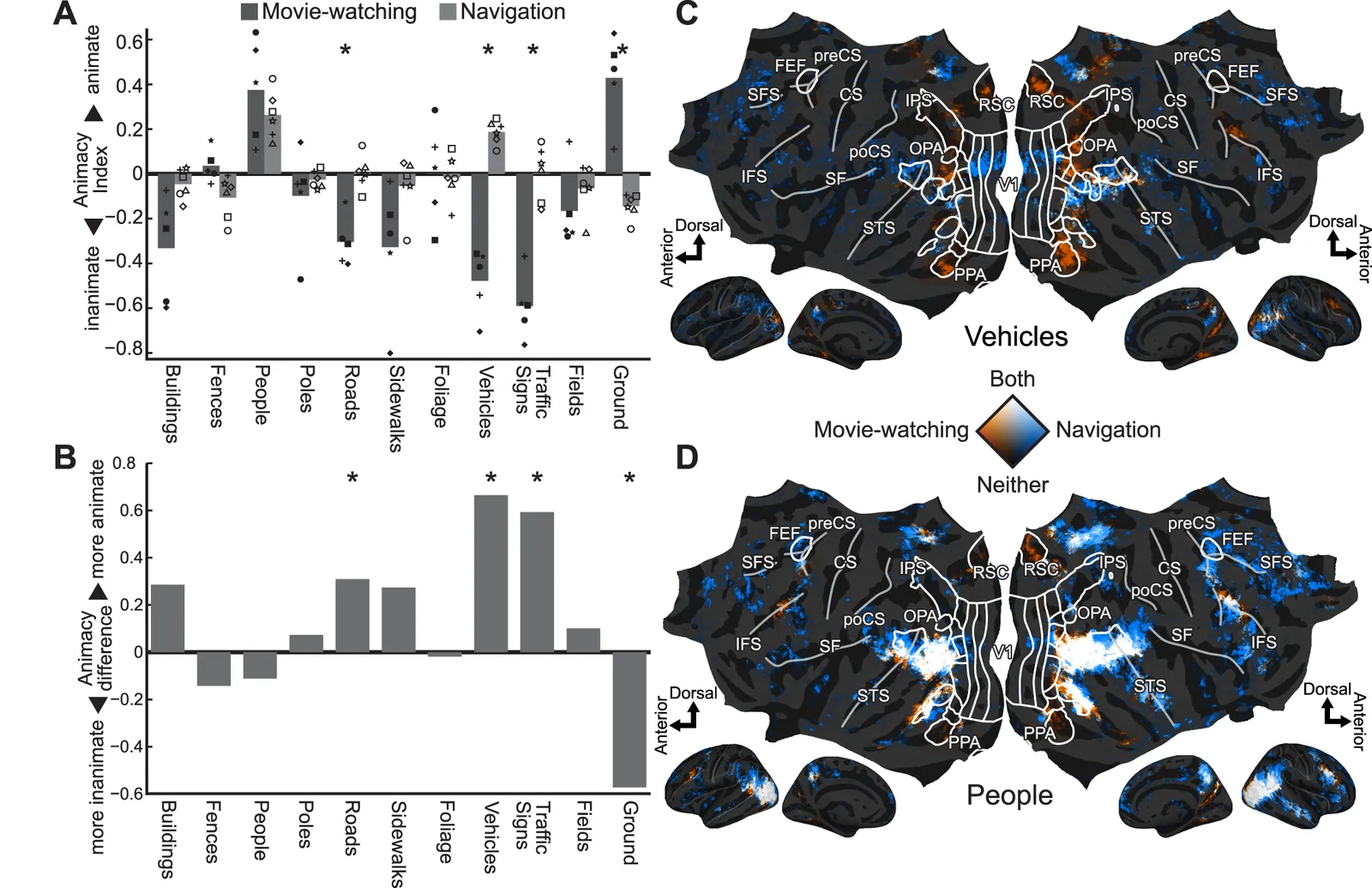
The animacy of object category representations differs between tasks. Animacy is a primary organizational principle for object category representations in the cortex. Because tuning shifts cause object categories to be represented by different cortical regions (Extended Data Fig. 5-1), this shift may correspond to changes in the animacy of object category representations. To quantify this, we defined an animacy index that quantified how object category representations project to template “animate” and “inanimate” representations. ***A***, The animacy indices for the representation of the 11 object categories are shown (bars indicate group average values while markers indicate individual participants). ***B***, The animacy of the representation of roads, vehicles, traffic signs, and the ground are different between the two tasks at the group level (FDR-corrected *p* < 0.05, permutation test). Notably, the representation of vehicles is inanimate during movie-watching, while animate during navigation. The representations for roads and traffic signs are more animate in navigation than in movie-watching, while the representation of ground shifts from animate to inanimate (see discussion on the change in the representation of “ground”). ***C***, Vehicles, the category with the strongest animacy difference, are represented in distinct cortical networks between navigation and movie-watching, whereas (***D***) people, whose animacy did not change, are represented in similar networks across tasks (for other categories, see Extended Data Fig. 5-2). These results show that one effect of task-related tuning shifts is a difference in the animacy of object category representations and that this difference is specific to task-relevant categories.

10.1523/ENEURO.0183-26.2026.f3-5 Figure 3-5 Average tuning shifts in the 11 individual ROIs in the cortical navigation network. The average shift (a.u.) for each category is shown for A) PPA-ColS, OPA-pLPC, and RSC-pPrCu, B) aLPC, aPrCu, and dPrCu, C) IFS and pSFS, and D) pmPFC, aIns, and FP. Bars show average shift across subjects, and dots indicate individual subjects. Download Figure 3-5, TIF file.

### Navigation-related regions show strong tuning shifts

In an earlier study, we reported that human navigation is supported by a cortical network of 11 different functionally distinct areas distributed across visual, parietal, and prefrontal regions of the brain (Zhang et al., 2025). This network represents a wide variety of perceptual, cognitive, and motor information directly relevant for navigation. If attention shifts tuning to increase the representation of task-relevant information, then functional areas comprising the navigation network should show large changes in visual-semantic tuning between passive movie-watching and navigation. We find that these 11 regions showed relatively strong differences in tuning between movie-watching and navigation (Fig. 2*C*). Nine regions (occipital areas PPA-ColS, OPA-pLPC, and RSC-pPrCu; parietal areas aLPC, aPrCu, and dPrCu; and prefrontal areas IFS, pmPFC, and pSFS) shift their visual-semantic tuning toward landmarks such as sidewalks, buildings, and fields, and away from vehicles and people. (The precise pattern of visual-semantic tuning shifts varies across these areas; see Extended Data Fig. 3-5 for details.) However, two areas within the navigation network (prefrontal areas aIns and FP) do not show a clear tuning shift direction. In sum, these results suggest that attention to task-relevant information changes visual-semantic representations broadly across task-relevant functional brain regions.

### Tuning shifts enhance navigation-relevant information

Tuning shifts serve to increase representation of task-relevant information. To identify the primary visual-semantic dimensions the brain prioritizes during navigation compared with movie-watching, we examined the loading of the 11 categories on the PCs of the tuning shifts (Fig. 4). PC 1 is an almost singular shift toward vehicles, suggesting the brain prioritizes the representation of other agents. PC 2 shows a shift toward buildings, ground, fields, foliage, and traffic signs, and a shift away from vehicles, roads, sidewalks, and people, suggesting that the brain seeks to better discriminate between scene identity-relevant categories and the entities that populate the scene. PC 3 shows a shift primarily toward people and away from roads, suggesting that the brain also seeks to better discriminate between navigational affordances and people. These results show that during navigation, the brain shifts its visual-semantic selectivity to better identify other agents in the environment and to discriminate these agents from navigational cues.

### Tuning shifts change the representation of object categories

These tuning shifts show that cortical regions change their visual-semantic selectivity between tasks. A possible effect is that each object category may therefore be represented by different functional networks across tasks. To test this hypothesis, we examined the spatial correlation of weights for each object category across the cortical surface between the movie-watching and navigation in each navigation participant. The representation of buildings, people, poles, roads, sidewalks, foliage, and vehicles were significantly different between movie-watching and navigation (*p* < 0.05, FDR corrected, bootstrap test; Extended Data Fig. 5-1). The difference in representation is not constant across categories: the representation of people is most correlated across tasks (*r* = 0.259 ± 0.075 mean ± SD across navigation participants) while the representation of vehicles is least correlated (*r* = −0.078 ± 0.085 mean ± SD across navigation participants). This reorganization suggests that across tasks, the brain redistributes object category representations across functional regions, with task-critical categories, e.g., vehicles, undergoing the biggest shift.

While direct comparison of weight vectors reveals that the brain systematically reorganizes object representations between tasks, it remains unclear whether these differences follow interpretable organizational principles. The animate-inanimate distinction is a primary organizing principle of object representations in the cortex (Naselaris et al., 2012; Grill-Spector and Weiner, 2014), and the perceived animacy of even simple geometric shapes can be affected by context such as their motion (Heider and Simmel, 1944). Thus, we investigated whether the cortex reorganizes object representations along this animacy dimension to reflect their behavioral relevance between tasks. Specifically, objects that appear to move intentionally might be represented in a more animate manner during navigation, even if they are typically considered inanimate objects. To quantify these differences, we defined an animacy index for the representation of object categories in each movie-watching and navigation participant. Results show that the animacy of roads, vehicles, traffic signs, and the ground differed significantly between the two experiments (FDR corrected *p* < 0.05, permutation test; Fig. 5*A*,*B*). The representation of vehicles showed the largest difference: vehicles are represented as inanimate objects during movie-watching but animate objects during navigation (FDR-corrected *p* < 5 × 10^−3^, permutation test). Roads and traffic sign representations are also more animate during navigation, while ground representation is more inanimate during navigation. To visualize these differences intuitively, object category representations were mapped onto the flattened cortical surface. Figure 5*C* shows the representation of vehicles, the category with the strongest animacy difference between tasks, while Figure 5*D* shows the representation of people, a category whose animacy did not change between tasks (for other categories, see Extended Data Fig. 5-2). Vehicle representations occupy distinct cortical networks between navigation and movie-watching, while people representations occupy similar networks across tasks. These results demonstrate that the brain flexibly reconfigures the perceived animacy of objects across task contexts.

10.1523/ENEURO.0183-26.2026.f5-1 Figure 5-1 Object representations in the cortex differ across tasks. As the cortex shifts its tuning, object categories become represented by different cortical networks. To quantify this difference, we computed the correlation between the weight vectors for each of the object categories between the two tasks. Bars show the group-average correlations for each category, and dots indicate individual subjects. Shaded regions indicate 95% confidence interval for the null distribution that there is no difference in the representation of each object category between navigation and movie-watching (for people and vehicles, the bottom of this confidence interval is above the upper bound of the plot). Stars indicate object categories that are significantly difference between the two tasks at the group level (FDR corrected p < 0.05, bootstrap test). The representations of buildings, people, poles, roads, sidewalks, foliage, and vehicles were significantly different between the two tasks. The difference in representation is not constant across categories: the representation of people is most similar between tasks, and the representation of vehicles is most dissimilar. These results suggest that task-related tuning shifts induce differences in the representations of object categories, and that these differences may be category-specific. Download Figure 5-1, TIF file.

10.1523/ENEURO.0183-26.2026.f5-2 Figure 5-2 Weight comparison for other object categories. Color scheme is the same as in Fig. 5C and D. Download Figure 5-2, TIF file.

## Discussion

To investigate tuning shifts between naturalistic tasks, we compared visual-semantic tuning in the human cerebral cortex measured during a passive movie-watching experiment and an active navigation experiment. Results show that visual-semantic tuning across the cerebral cortex differs significantly between the two experiments. The magnitude of the shifts in visual-semantic tuning varies across ROIs; the strongest shifts are found in regions that represent information about navigation and spatial attention. These results suggest that many visually selective regions of the brain shift their tuning between tasks.

The matched-filter theory (Mazer and Gallant, 2003; David et al., 2008; Çukur et al., 2013) of attention predicts that the cortex shifts its tuning between tasks to enhance the representation of task-relevant information. In contrast, a purely gain-based account of attention predicts a uniform scaling of responses across stimuli within each voxel (Treue and Martínez Trujillo, 1999), and a sharpening-based account of attention predicts a suppression of nonpreferred object categories but no change in the overall direction of the tuning vector (Martinez-Trujillo and Treue, 2004). The tuning differences between tasks that we report here are most consistent with the matched-filter theory; they cannot be explained by attention-based changes in gain or sharpening. Neurophysiology studies in primates have shown that tuning of neurons in the prefrontal and anterior-ventral temporal cortex is task dependent (Asaad et al., 2000; Johnston and Everling, 2006; Koida and Komatsu, 2007; Warden and Miller, 2010; McKee et al., 2014). Our results suggest that these changes occur broadly across the human brain, including in category-selective regions within the anterior visual cortex.

The direction of the tuning shift between tasks differed systematically across functional regions. During active navigation, the precuneus, TPJ, and prefrontal cortex shifted their tuning toward the vehicles. These regions have been implicated in theory-of-mind and higher-level planning (Koechlin et al., 2000; Saxe and Kanwisher, 2003; Kaller et al., 2011), suggesting that these shifts may support cognitive processes involved in predicting the actions of other agents during navigation. On the other hand, RSC, OPA, and PPA, three scene-selective regions, and IPS and FEF, two visual attention regions, shifted their tuning toward traffic signs and poles. This pattern likely reflects the relatively greater behavioral importance of traffic signs during navigation. These distinct tuning shifts between functional regions suggest that different cognitive systems are used to represent other agents and to represent navigational rules.

These tuning shifts are accompanied by a remarkable change in the animacy of vehicle representations: vehicles are represented as inanimate objects during movie-watching, but as animate objects during navigation. While it has long been known that the motion of geometric objects can influence their perceived animacy (Heider and Simmel, 1944; Santos et al., 2008, 2010; Osaka et al., 2012; Schultz and Bülthoff, 2019), this result shows that the animacy of the same real-world object category differs across naturalistic tasks. Because the virtual car models used in our study did not include visible drivers, this shift cannot be attributed to the co-occurrence of drivers with vehicles. Instead, it is likely related to task context. During movie-watching, vehicles are merely human-operated conveyances, but during navigation participants are driving and other vehicles are peer agents. Another unexpected finding in our study is that roads and traffic signs also shifted toward more animate representations during navigation, despite lacking agency. We speculate that animacy might reflect an object's capacity to influence behavior rather than biological agency per se: a speed limit sign, like another vehicle, directly constrains participants' actions during navigation.

The distinct tuning shifts observed across functional regions contrast with previous work that examined visual-semantic tuning shifts within a single task (Çukur et al., 2013; Shahdloo et al., 2022). Those studies found that tuning across the cortex shifts broadly in the direction of the attended category. The between-task tuning shifts that we observe here are much more spatially heterogeneous than those reported within tasks, suggesting a more substantial reorganization of functional networks. In sum, our results suggest that the matched-filter mechanism may enhance task performance not only at the level of individual voxels, but also at the level of large-scale networks.

The data compared in this study were collected in two separate experiments that were not originally designed to be compared with one another, and so they differ in other respects beyond the task, including the stimuli used and participant groups. The possibility that these other uncontrolled differences also contributed to the observed tuning differences could not fully be excluded. However, control analyses suggest that stimulus differences are unlikely to account for the observed tuning shifts. To compare across participant groups, the group-average weights from the movie-watching participants were mapped to the navigation participants' cortical surfaces for comparison. Previous studies had shown that the first 5–6 semantic PCs are consistent across participants (Huth et al., 2012, 2016; Deniz et al., 2019) and that the group-average model explains ∼78% of the variance in individual participants (Visconti di Oleggio Castello et al., 2025). These results suggest that the movie-watching group model provides a reasonable baseline for navigation participants, though some individual variance is inevitably lost. Despite these differences, the systematic visual-semantic tuning shifts observed correspond well to task context, suggesting that they are more likely task-related rather than driven by uncontrolled confounds.

One specific minor confound in this study is that statistics of the “ground” label differed between the movie-watching experiment and the navigation experiment. In the movie-watching experiment, the movies were hand-tagged by an observer who selected two to four of the most salient object categories that appeared in each second of the movies. Inspection of the tags revealed that the ground was not always labeled, despite being present in many scenes. Instead, the ground was often labeled with environmental descriptors such as “tundra” or “savannah” that cooccurred with animals. This bias likely caused the animacy analysis to show that the ground was represented as an animate category in the movie-watching experiment (Fig. 5*A*). This bias did not arise in the navigation task, as semantic segmentation was performed by the game engine, which separated ground labels from animate agents. The animacy of the ground category should therefore be interpreted with caution in the movie-watching experiment due to this labeling confound.

The results reported here extend previous work that examined how attention shifts visual-semantic tuning across the human cortex. While previous studies have shown that attention shifts visual-semantic tuning in the human brain toward attended visual-semantic concepts (Çukur et al., 2013; Harel et al., 2014; McKee et al., 2014; Nastase et al., 2017; Shahdloo et al., 2022), our comparison of visual-semantic tuning between naturalistic tasks points to tuning shifts across tasks with distinct behavioral demands. Because this comparison drew on data from two separate experiments that differed beyond tasks, future targeted studies that control for more factors, such as within-subject comparisons and matched stimuli between tasks, will more precisely characterize task-related tuning shifts. Taken together, these results suggest that the human brain dynamically optimizes its limited neural resources toward task-relevant information, enabling it to efficiently perform diverse tasks across complex, ever-changing environments.

## Synthesis

Reviewing Editor: Niko Busch, Westfalische Wilhelms-Universitat Munster Decisions are customarily a result of the Reviewing Editor and the peer reviewers coming together and discussing their recommendations until a consensus is reached. When revisions are invited, a fact-based synthesis statement explaining their decision and outlining what is needed to prepare a revision will be listed below. The following reviewer(s) agreed to reveal their identity: NONE. Note: If this manuscript was transferred from JNeurosci and a decision was made to accept the manuscript without peer review, a synthesis may not be available.

# Review synthesis

This manuscript was evaluated by two expert reviewers, one of whom had reviewed a previous version of the manuscript. Reviewer 2 is satisfied with the revisions and recommends acceptance. Reviewer 1, who is new to this manuscript, acknowledges the quality of the work but raises thoughtful concerns about the framing: specifically, they suggest recasting the study as an exploratory comparison rather than a hypothesis-driven investigation, and recommend more explicitly acknowledging the many uncontrolled differences between the two tasks as limitations. I find these comments reasonable and constructive, as they do not challenge the analyses or results but call for a more accurate characterization of what the study can and cannot conclude. I therefore recommend revisions that involve reframing the study and expanding the discussion to discuss these limitations.
