# Supplementary material for: Visual-Semantic Tuning across the Cortex Shifts between Tasks
Source: eNeuro. 2026 Sep 8;13(9):ENEURO.0183-26.2026. doi: 10.1523/ENEURO.0183-26.2026 (PMC13583329; doi:10.1523/ENEURO.0183-26.2026)
Supplement: Supplementary file 1 [file eneuro-13-ENEURO.0183-26.2026-s011.docx]

| Feature Space | Short Description |
| --- | --- |
| Eyetracking power | The aggregate power across channels encoding gaze location and its derivatives in screen space encoded in cartesian and polar coordinates |
| Gaze grid | Gaze location in screen space encoded with a hexagonal grid basis (Nau et al., 2018) |
| Framewise motion-energy | Spatiotemporal Gabor energy in screen coordinates (Nishimoto and Gallant, 2011) |
| Retinotopic motion-energy | Spatiotemporal Gabor energy in retinotopic coordinates |
| Frame semantics | Semantic content of the screen |
| Depth | Distance to objects in the virtual world |
| Scene structure | The distribution of visible surfaces and their orientations relative to the participant’s viewpoint (Lescroart and Gallant, 2019) |
| Spatial semantics | Visible semantic content of the virtual world around the participant in egocentric coordinates |
| Navigational affordance | Where in the scene that the participant can move through (Bonner and Epstein, 2017) |
| Controls | Steering, gas, brake, and gear selections from the subject |
| Future path | Future path that the participant will take in egocentric coordinates |
| Distance to next turn | Fraction of total distance travelled until the next turn encoded with a Fourier basis (Nitz, 2006) |
| Time to next turn | Fraction of total time travelled until the next turn encoded with a Fourier basis |
| Beeline distance remaining | Beeline distance remaining to the destination (Howard et al., 2014) |
| Path distance remaining | Distance remaining along the actual planned path to the destination (Howard et al., 2014) |
| Spatial route progression (continuous) | Fraction of total distance travelled to the destination encoded by a Fourier basis (Alexander and Nitz, 2017) |
| Temporal route progression (continuous) | Fraction of total time travelled to the destination encoded by a Fourier basis |
| Spatial route progression (binned) | Fraction of total distance travelled to the destination encoded by a set of indicator features |
| Temporal route progression (binned) | Fraction of total time to the destination encoded by a set of indicator features |
| Destination vector | An egocentric vector to the destination (Howard et al., 2014; Brown et al., 2016; Ormond and O’Keefe, 2022) |
| Destination-anchored vector | An allocentric vector from the destination to the current location of the participant |
| Destination grid representation | Position of the destination in the world encoded with a hexagonal grid basis |
| Allocentric path integration | Allocentric vector to the start location of the current trial (Sherrill et al., 2013; Chrastil et al., 2015) |
| Egocentric path integration | Egocentric vector to the start location of the current trial |
| Beeline distance elapsed | Beeline distance from the start of the current trial (Sherrill et al., 2013; Chrastil et al., 2015) |
| Path distance elapsed | Actual path distance travelled from the start of the current trial |
| Time elapsed | Time elapsed since the start of the current trial encoded by a set of indicator features |
| Time elapsed phase | Time elapsed since the start of the current trial encoded by a Fourier basis |
| Place fields | Place field-like representations of the participant’s 2D location in the virtual world(O’Keefe and Dostrovsky, 1971; Ekstrom et al., 2003) |
| Grid cells | Features with grid cell-like periodic spatial selectivity on a hexagonal grid in the virtual world (Fyhn et al., 2004; Sargolini et al., 2006) |
| Head direction | The head direction of the participant in the world encoded with a set of indicator bins (Taube et al., 1990; Vass and Epstein, 2013) |
| 6-fold Head direction | The head direction of the participant in the world, with 6-fold symmetry, encoded with a quadrature filter (Doeller et al., 2010) |
| Gaze direction | The gaze direction of the participant in the world relative to the current heading direction encoded with a set of indicator bins |
| 6-fold gaze direction | The gaze direction of the participant in the world relative to the current heading direction, with 6-fold symmetry, encoded with a quadrature filter |
| Road graph | The planned path of the subject expressed as a set of edges on a graphical representation of the road network (Chrastil and Warren, 2014; Javadi et al., 2017; Warren et al., 2017) |
| Pedestrians | Positions of pedestrians in egocentric coordinates (Danjo et al., 2018; Omer et al., 2018) |
| Vehicles | Positions of other cars in egocentric coordinates |
